# Celiac Disease in Conjunction with Hereditary Fructose Intolerance as a Rare Cause of Liver Steatosis with Mild Hypertransaminasemia—A Case Report

**DOI:** 10.3390/pediatric13040070

**Published:** 2021-11-01

**Authors:** Anna Bobrus-Chociej, Agnieszka Pollak, Natalia Kopiczko, Marta Flisiak-Jackiewicz, Rafał Płoski, Dariusz M. Lebensztejn

**Affiliations:** 1 Department of Pediatrics, Gastroenterology, Hepatology, Nutrition and Allergology, Medical University of Białystok, Jana Kilińskiego 1, 15-089 Białystok, Poland; nwasilewska@interia.pl (N.K.); m_flisiak@op.pl (M.F.-J.); lebensztejn@hoga.pl (D.M.L.); 2 Department of Medical Genetics, Medical University of Warsaw, Żwirki i Wigury 61, 02-091 Warsaw, Poland; agnieszka.pollak@wum.edu.pl (A.P.); rafal.ploski@wum.edu.pl (R.P.)

**Keywords:** celiac disease, hereditary fructose intolerance, liver steatosis, hypertransaminasemia

## Abstract

Celiac disease (CD) has been associated with several genetic and autoimmune disorders, but its association with hereditary fructose intolerance (HFI) is very rare. The possibility of an association between CD and HFI should be considered, especially in patients with a lack of improvement after a gluten-free diet. Children with HFI often present with a wide range of symptoms, however, data about a strong aversion to fruits and sweets may be helpful to establish the diagnosis. The diagnosis of HFI should be confirmed in genetic testing. Both CD and HFI may present with liver steatosis with hypertransaminasemia. In patients with these two disorders, the dietary restrictions of gluten and fructose improve clinical symptoms and protect them from secondary complications. We report the case of a child with the concurrence of these two disorders.

## 1. Introduction

Celiac disease (CD) is a systemic, immune-mediated disorder elicited by gluten in genetically susceptible individuals. The risk of developing CD is increased by certain variants of the HLA-DQ2 and HLA-DQ8 genes. In addition, there are at least three different versions of the HLA-DQ2 gene. The disease presents with a variety of gastrointestinal and extra-intestinal symptoms, e.g., liver pathology (steatosis and/or hypertransaminasemia) [1]. The endoscopic biopsy of small bowel shows varying degree of villus atrophy, mucosal inflammation and crypt hyperplasia. The treatment is a complete elimination of gluten from the diet, and results in most patients with clinical response and histologic improvement [1,2]. CD has been associated with several genetic and autoimmune disorders, e.g., type 1 diabetes mellitus, rheumatoid arthritis or autoimmune thyroid disease. The associated illnesses may appear both at the time of diagnosis of celiac disease and throughout the evolution of the disease [3]. However, coexistence of celiac disease and hereditary fructose intolerance (HFI) seems to be extremely rare.

HFI is a rare autosomal recessive genetic disorder characterized by the deficiency of aldolase B activity, resulting in fructose accumulation in the liver, kidney and intestine. The disease is caused by mutation of the gene for aldolase-B (ALDOB) on chromosome9q22.3. At present, over 40 causative mutations of the ALDOB have been documented. Persistent fructose ingestion can lead to hepatic steatosis, fibrosis and cirrhosis and signs of proximal renal tubular dysfunction. HFI is believed to be underdiagnosed because of its wide spectrum of clinical signs [4,5,6]. The incidence of the disease ranges from 1:20,000 to 1:60,000 [5]. Some patients remain undiagnosed due to the voluntary avoidance of sweet foods [4].

Here we present the case of a child with concurrence of celiac disease and hereditary fructose intolerance.

## 2. Case Presentation

A 5-year-old girl was admitted to the Department of Pediatrics, Gastroenterology, Hepatology, Nutrition and Allergology, Medical University of Białystok because of abdominal pain and failure to thrive. Parameters of her physical development were decreasing: body mass 17.4 kg (10–25c), height 114 cm (25c), BMI 13.5 kg/m^2^ (3–10c). Physical examination showed abdominal distention. Laboratory tests revealed increased activity of aminotransferases: ALT 50 U/L (norm: 37 U/L), AST 44 U/L (norm: <40 U/L), low concentration of albumin and vitamin D in serum. Urinalysis did not show abnormalities. Abdominal ultrasonography revealed slightly enlarged liver with increased echogenicity (steatosis). Different causes of elevated aminotransferases were excluded: viral (e.g., HBV, HCV, CMV), toxic, selected metabolic disorders (alpha-1- antitrypsin deficiency, Wilsons’ disease) and autoimmune hepatitis.

On investigation, anti-tissue transglutaminase (a-tTGA) IgA antibodies were positive: 292 U/mL (cut off upper limit: <10 U/mL). Total serum IgA concentration was normal. The anti-endomysial antibodies (EmA) IgA test was also positive. Since a-tTGA IgA value was 10 times the upper limit of normal, a no-biopsy approach was recommended according to the guidelines [1]. However, parents did not accept this decision and endoscopy was performed. Gastroduodenoscopy showed fissures on tops of the duodenal folds. Duodenal biopsy confirmed subtotal villus atrophy with crypt hyperplasia and increased intraepithelial lymphocytes (Marsh IIIb). Genetic testing was positive for HLA DQ2. Soon after CD was diagnosed, the child was put on a gluten-free diet with slight improvement. Abdominal pain and diarrhea resolved after eight weeks, however, the BMI remained on the same level (3–10c).

To investigate the maintenance of symptoms (poor appetite, failure to thrive, liver steatosis, hypertransaminasemia) despite eight weeks of gluten-free diet, additional examination was performed. Reviewing the dietary history intake, we found that the gluten exclusion was very strict, however the child refused to eat sweets, certain fruits and vegetables. Suspecting HFI, the pattern of transferrin isoforms in serum was performed and the result was abnormal: asjalotransferin—1.3% (norm: 0), monosjalotransferrin—0.6% (norm: 0), disjalotransferrin—11.4% (norm: 1.5–6.2), trisjalotransferrin—9.3% (norm: 7.4–17.1), tetrasjalotransferrin—53.5% (norm: 55.7–67.2), pentasjalotransferrin—19.7% (norm: 13.2–19.9), hexasjalotransferrin—4.2% (norm: 2.5–5.6).

For that reason, the patient was initiated on a fructose-free diet. A gluten-free diet was continued and follow-up visits showed good catch-up growth. Aminotransferases normalized after two months and control USG showed decreased grade of liver steatosis. Elastography (Fibroscan) showed normal liver stiffness. HFI was confirmed in a genetic test.

**Figure 1:**
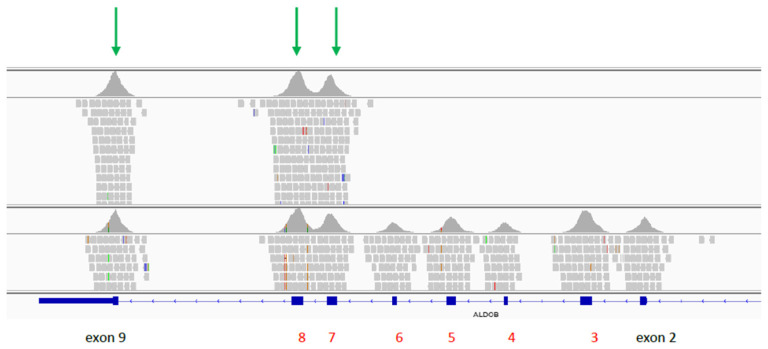
Graphical presentation of the partial deletion of ALDOB gene created in Integrative Genomics Viewer v.2.8. Upper panel represents proband, lower panel represents control sample. Green arrows denote remaining exons of ALDOB gene.

DNA from the girl and her parents was isolated from peripheral blood according to standard protocol. Initial diagnostic approach was based on Sanger sequencing of the coding part of the ALDOB gene. During the procedure, no amplification of the exon 2–6 was denoted on the proband’s DNA, despite its high quality (established by the amplification of the first and 7–9 exons of ALDOB gene), which suggested homozygous partial deletion of the ALDOB gene. To verify the presence of the deletion, and exclude other causative genes, next generation sequencing was performed on the proband’s DNA sample with the SeqCap EZ 1000 genes probes (Roche, Basel, Switzerland)— a custom panel targeting the coding part of 1000 genes causative for Mendelian disorders (including the ALDOB gene). Subsequently, paired end index sequencing (2 × 100) was performed on the HiSeq 1500 sequencer (Illumina Inc., San Diego, CA, USA) to obtain 14,073,559 reads (100% of target bases were covered at a minimum of 20×, whereas 100% had coverage of min. 10×). The obtained raw data were processed with the pipeline described previously [7]. Obtained results were inspected with an Integrative Genomics Viewer (IGV). The results confirmed the presence of homozygous deletion of exon 2–6 within the ALDOB gene (Figure 1). Furthermore, Real-Time PCR assay of the fifth exon of the ALDOB gene (with SYBR Green) was performed to confirm the carrier status of the parents. The difference between cycle threshold (ΔCt) for amplification curves was one, (compared to the control samples without deletion) which confirms the presence of the heterozygous deletion in both parents.

Control elastography (Fibroscan) showed normal liver stiffness (E 6.8 kPa).

## 3. Discussion

Coexistence of celiac disease and hereditary fructose intolerance (HFI) seems to be a rare association. The main cause of failure to respond to a gluten-free diet in patients with celiac disease is gluten contamination [8]. However, in cases of strict gluten elimination, associated conditions should be considered. Celiac disease may coexist with several genetic and autoimmune diseases also involving liver abnormalities [9,10]. Celiac hepatitis, characterized by gluten-responsive mild elevation of transaminases, is the most common liver manifestation of celiac disease [11], followed by “bright liver” in an ultrasound [9]. Shared autoimmunity predisposition and increased intestinal permeability have been suggested to be the most relevant events in the pathogenesis of liver injury in celiac disease [12].

HFI is an under-diagnosed, preventable, life-threatening condition. The disorder is caused by the deficiency of the aldolase B enzyme (fructose 1,6- biphosphate aldolase), encoded by the ALDOB gene. The enzyme plays a very important role in the control of the metabolism of fructose and glucose, regulating both glycolysis and gluconeogenesis [13]. Following dietary exposure to fructose, sucrose or sorbitol typical clinical symptoms (nausea, vomiting, chronic growth restriction) and metabolic disturbances (hypoglycemia, lactic acidemia, hyperuricemia) occur. HFI typically first manifests when fructose-containing foods are introduced in the course of weaning infants from breast milk. The infants may acutely develop lethargy, seizures and coma. Untreated HFI may cause renal and hepatic failure. The early diagnosis allows dietary treatment which consists mainly of a life-long diet free from fructose, sucrose and sorbitol, and the long-term prognosis is good [14].

In case of our patient a gluten-free diet in treating celiac disease resulted in slight improvement of clinical symptoms. Strong aversion to sweets found in the dietary history raised the suspicion of coexisting hereditary fructose intolerance, and the disease was confirmed in a genetic test.

To the best of our knowledge, there are only few case reports presenting patients of coexisting CD and HFI [15,16,17]. Bharadia et al. [15] reported a case of a 22-month-old boy with recurrent diarrhea and vomiting. Duodenal biopsy was assessed as Marsh IIIa (partial villous atrophy, crypt hyperplasia and increased intraepithelial lymphocytes). A gluten- free diet led to partial improvement after 4 months. Careful dietary history revealed a strong aversion to sweets. In laboratory tests, activity of aminotransferases was elevated and an oral fructose test caused vomiting. Liver biopsy showed microvesicular steatosis and established cirrhosis. A strict fructose-free diet and continuation of gluten-free diet improved the child’s condition and physical development. In this patient, a genetic test for HFI was not performed.

Pacurar et al. [16] presented a case of a 5-year-old boy, known with celiac disease, admitted to the Intensive Care Unit with a coma of unknown etiology. The initial diagnosis was Reye’s syndrome. Laboratory findings showed very high transaminases (>8 × UNL) and hypoglycemia. Abdominal ultrasound reveled an increased size of the liver and diffuse liver steatosis. Reviewing the dietary history intake, the authors found that the patient refused to eat certain fruits, vegetables and sweets, presenting vomiting after ingestion of small amounts of these foods. Using polymerase chain reaction (PCR) it was established that the boy was homozygous for mutations known as p.Ala174Asp (HFI). The child was initiated on a fructose- and gluten-free diet, and follow-up visits showed good catch up growth, and a complete recovery of the liver disease.

In our study, next generation sequencing was used to confirm HFI in the patient and the Real-Time PCR assay of the fifth exon of ALDOB gene was performed to confirm the carrier status of the parents.

Bhardia et al. [15] and Pacurar et al. [16] performed a liver biopsy in their patients and histopathological examination showed non-specific micro-vesicular steatosis. However, liver biopsy is not recommended as a diagnostic procedure in HFI, and our patient did not meet the criteria to perform a liver biopsy [13,18].

In the above-mentioned cases, likewise in our patient, HFI was suspected due to the poor improvement of CD on a gluten-free diet and aversion to sweets. However, Ciacci et al. conducted an inverse analysis. They examined 38 Italian patients with HFI with persisting symptoms, notwithstanding a fructose-free diet. The authors identified CD in four of them; three of these patients were children. The incidence of CD in their group of HFI patients was higher (10%) than in the general population (1%) [17].

What is more, HFI and CD are listed among less-common factors of hepatic steatosis, especially in non-obese children, therefore these conditions should be considered in lean patients with liver steatosis [12,19].

## 4. Conclusions

Patients with celiac disease with persisting symptoms despite a gluten-free diet should be tested for possible association with other genetic and immune disorders.

Children with hereditary fructose intolerance often present with a wide range of symptoms, however, data about a strong aversion to fruits and sweets may be helpful to establish the diagnosis.

In celiac disease associated with hereditary fructose intolerance, a restricted gluten- and fructose-free diet improves clinical symptoms and prevents secondary complications.

## Data Availability

Via email from corresponding author.
